# Supplementary material for: Dynamic plasmonic colour display
Source: Nat Commun. 2017 Feb 24;8:14606. doi: 10.1038/ncomms14606 (PMC5333121; doi:10.1038/ncomms14606)
Supplement: Supplementary Information — Supplementary Figures and Supplementary Notes [file ncomms14606-s1.pdf]

Enlarged SEM images

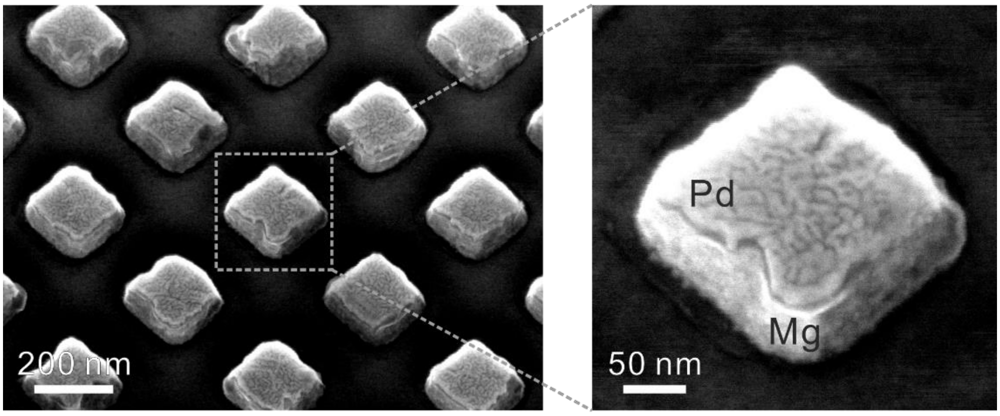

Supplementary Figure 1 | Enlarged SEM images of the representative Mg nanostructures.

Supplementary Note 1 | Spectral analysis

The analytical dispersion curves of the RAs are given by

$$\lambda_{\mathrm{RA}} = \frac{(s+d)}{\mathrm{m}}\sqrt{\varepsilon_d} \tag{1}$$

where ε_d is the permittivity of the corresponding dielectric layer, m is an integer, signifying the order of the resonance, and *s*+*d* is the periodicity of the array. The LSPR positions were calculated using FEM simulations based on single particles.

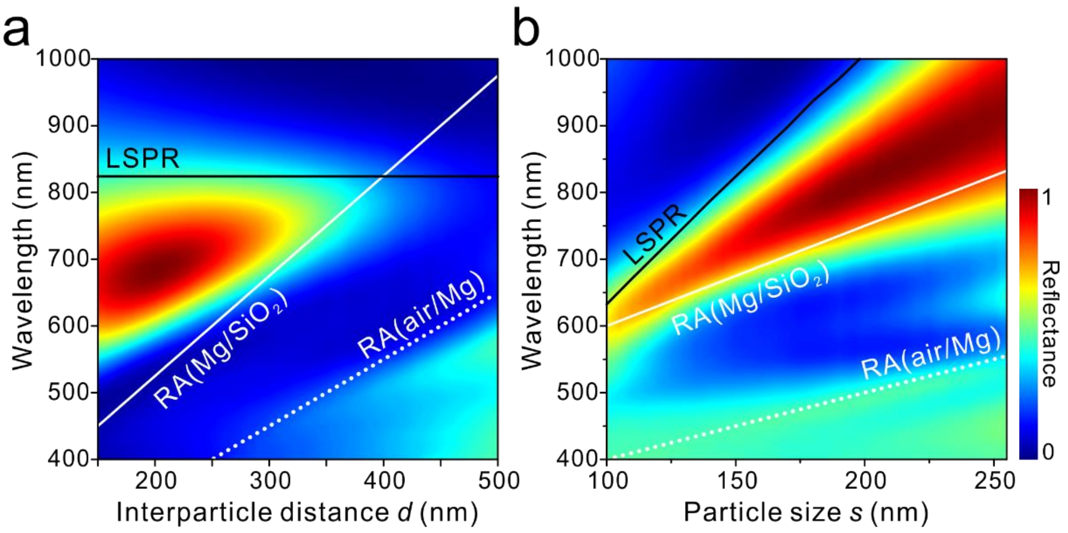

Supplementary Figure 2 | (a) Simulated contour map of the reflectance peak positions in dependence on *d* (*s* = 150 nm). (b) Simulated contour map of the reflectance peak positions in dependence on *s* (*d* = 300 nm). The black line indicates the LSPR modes. The white-solid and white-dotted lines indicate the RAs for the Mg/SiO_2 and air/Mg interfaces, respectively.

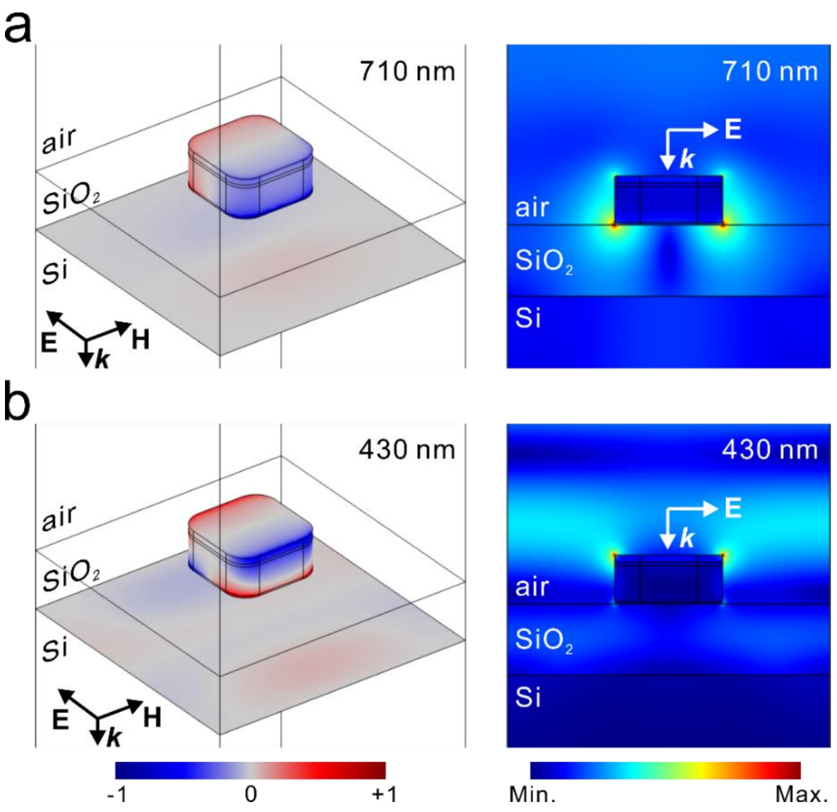

Supplementary Figure 3 | (a) Simulated charge and electric field distributions at 710 nm (a) and 430 nm (b). *s* = 150 nm and *d* = 300 nm.

Dynamic optical spectra recorded during hydrogenation and dehydrogenation

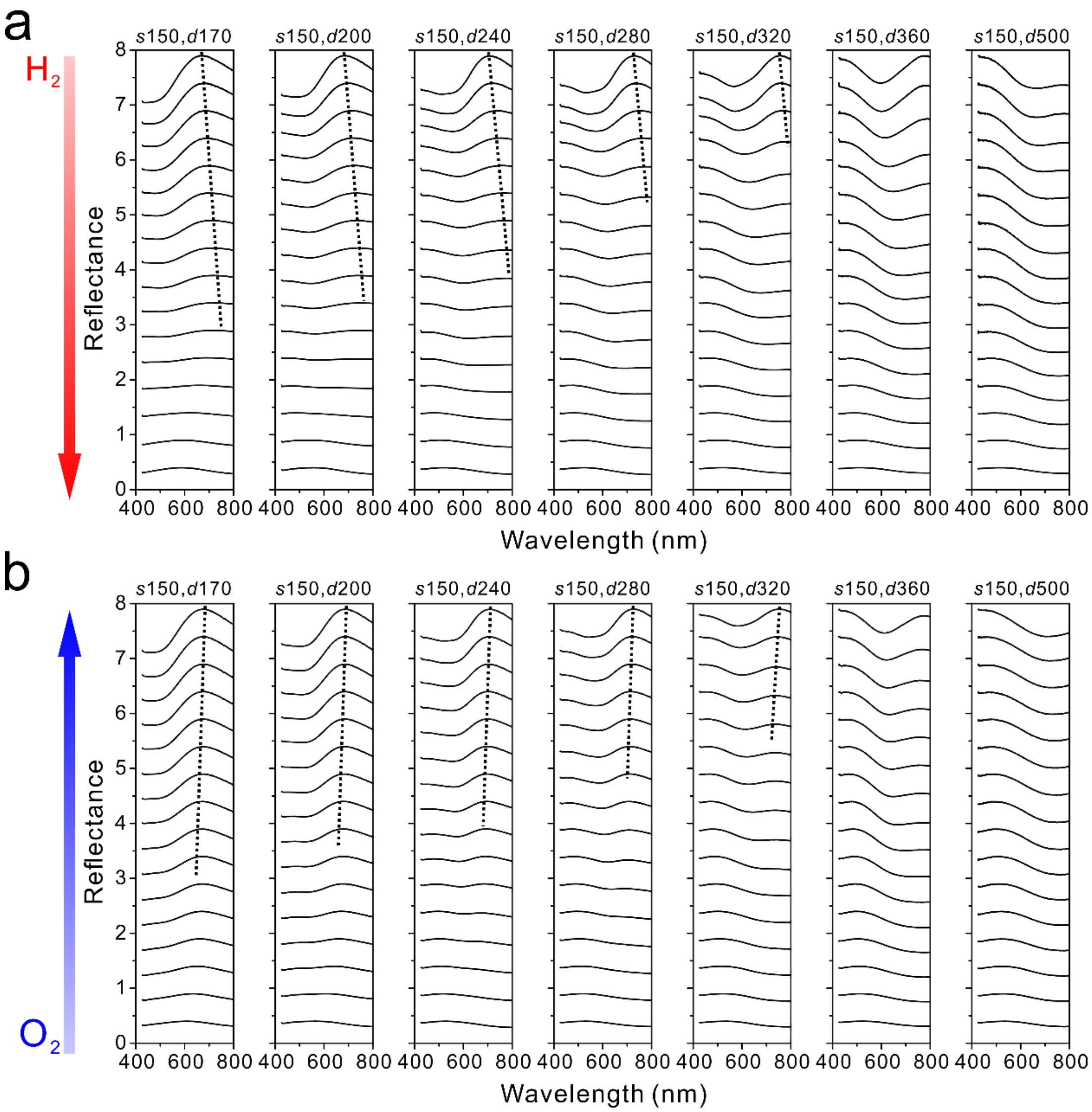

Supplementary Figure 4 | Representative dynamic reflectance spectra recorded during hydrogen (a) and oxygen exposure (b). The dotted lines indicate the positions of the reflectance peaks.

Simulated reflectance spectra for the representative structures in the hydrogenated state

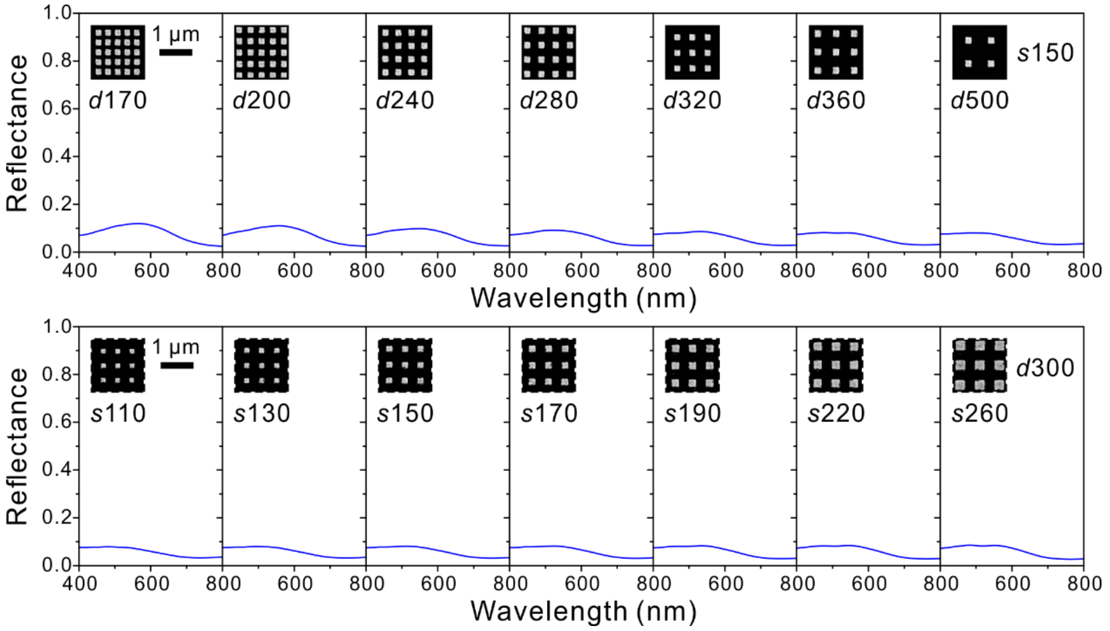

Supplementary Figure 5 | Simulated reflectance spectra of the colour squares in Fig. 1c after hydrogenation.

Illumination intensity

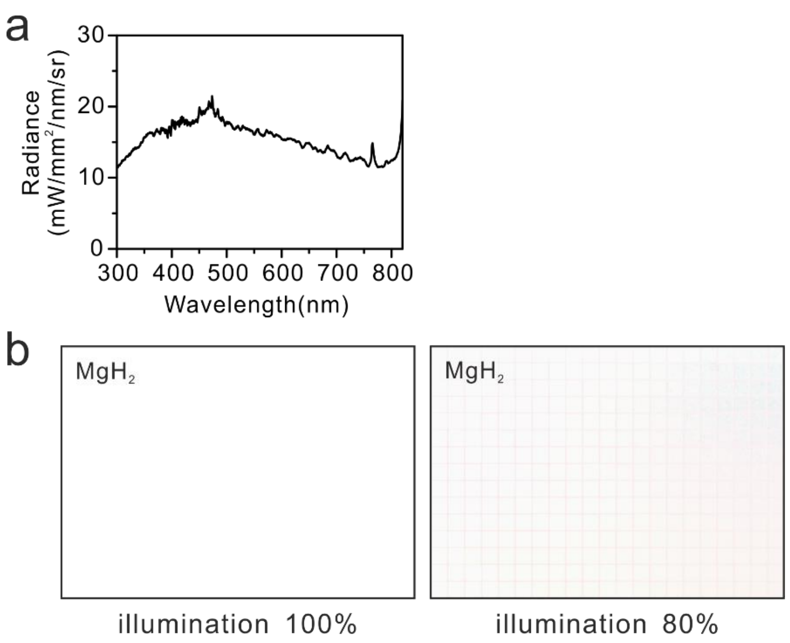

Supplementary Figure 6 | (a) Spectral radiance of the EQ-99 white light source. (b) Optical images of the colour palette after hydrogenation under different illumination intensities. When the illumination intensity is reduced from the full power (100%) to a lower value (80%), colours are observed at areas with large pitches. This issue can be solved by creating individual colour elements with pitches all no larger than 250 nm.

CIE chromaticity diagram analysis

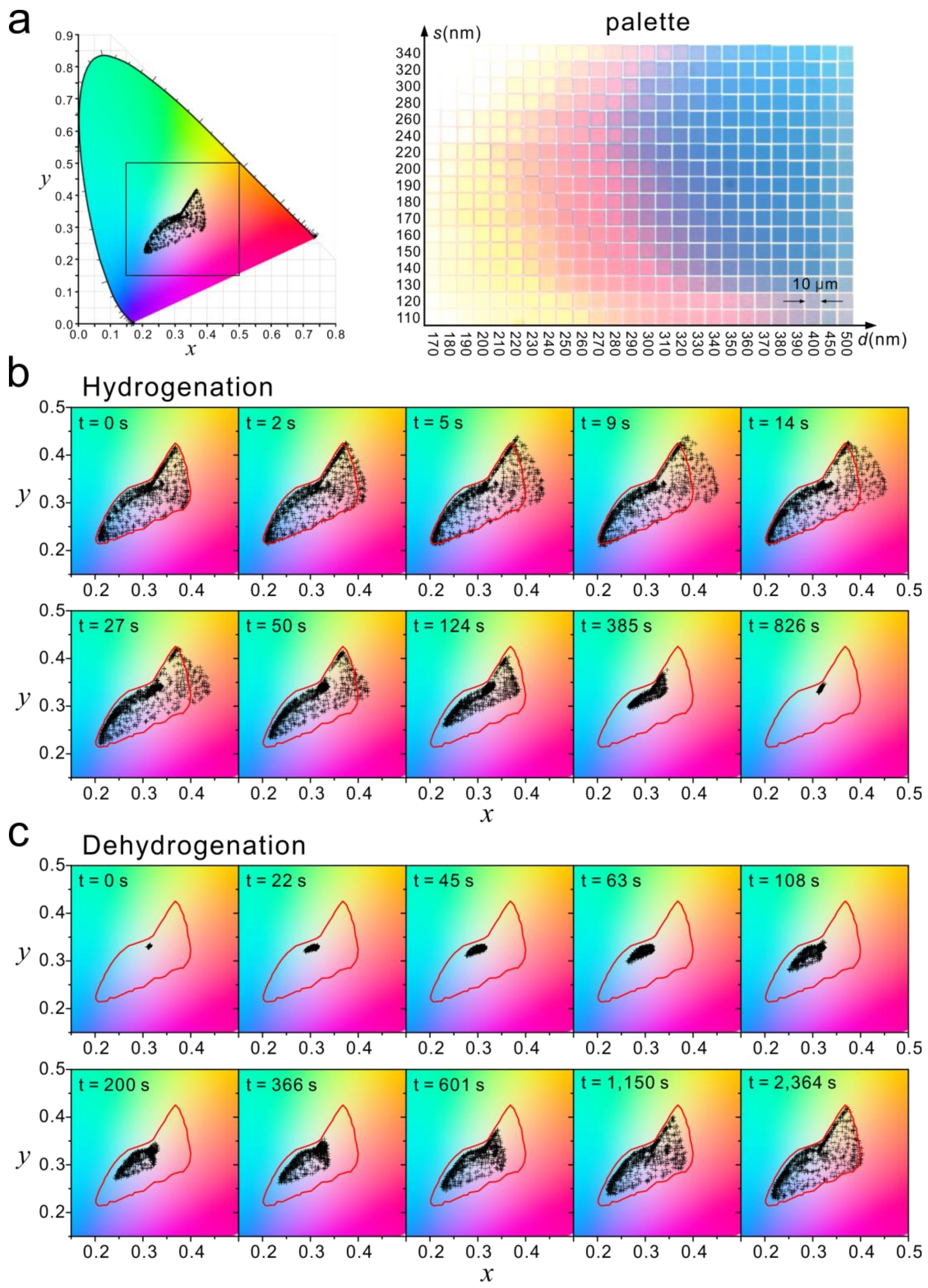

Supplementary Figure 7 | (a) CIE 1931 chromaticity diagram overlaid with all the colours from the palette. The CIE illuminant D65 is used for the conversion to the chromaticity coordinate. Each black cross in the CIE map indicates a colour square from the palette. (b) Evolution of all the palette colours during hydrogenation. Red lines indicate the original colour range. New colours are generated during hydrogenation between t = 0 *s* and t = 50 *s*. (c) Evolution of all the palette colours during dehydrogenation.

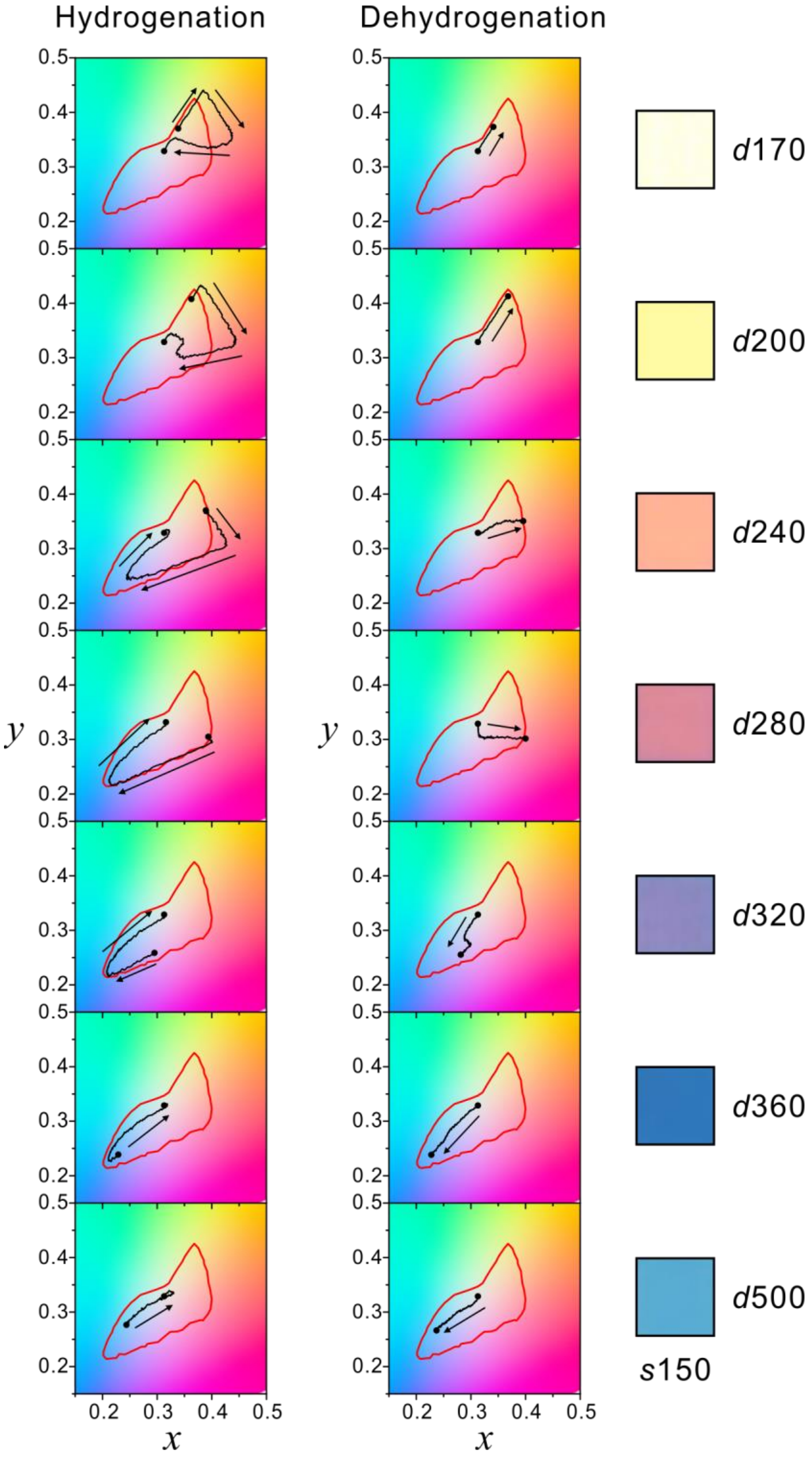

Supplementary Figure 8 | CIE 1931 chromaticity coordinate for the selected seven colours in Fig.1c. Black lines represent the pathways of the colour changes during hydrogenation /dehydrogenation.

Supplementary Note 2 | Layout generation

Red, green, and blue (RGB) values for each colour square were extracted from the optical micrograph of the palette in Fig. 1b, and this serves as a colour database. Any arbitrary image can be pixelated using a code written in MATLAB. The code extracts the RGB values for each pixel and finds a closest match to a certain (*s*, *d*) from the palette using a CIE L*a*b* space least distance method based on the colour database.

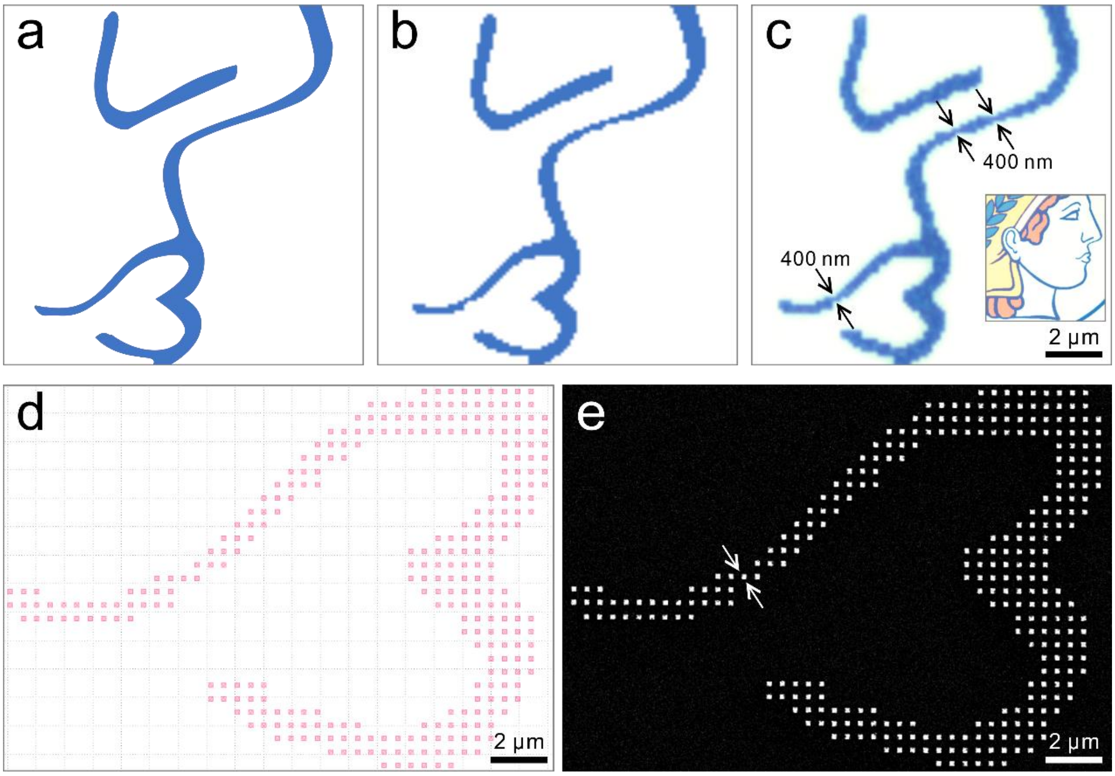

Supplementary Figure 9 | (a) Finest part of the MPG's Minerva logo in Fig. 2. (b) Corresponding pixelated image. (c) Optical micrograph of the logo. A 50 × objective (NA = 0.55) was used in this case. The overview image is shown as the inset. (d) Corresponding layout. (e) SEM image of the logo. The narrowest part consists of only one Mg nanoparticle.

NA dependence and angle dependence

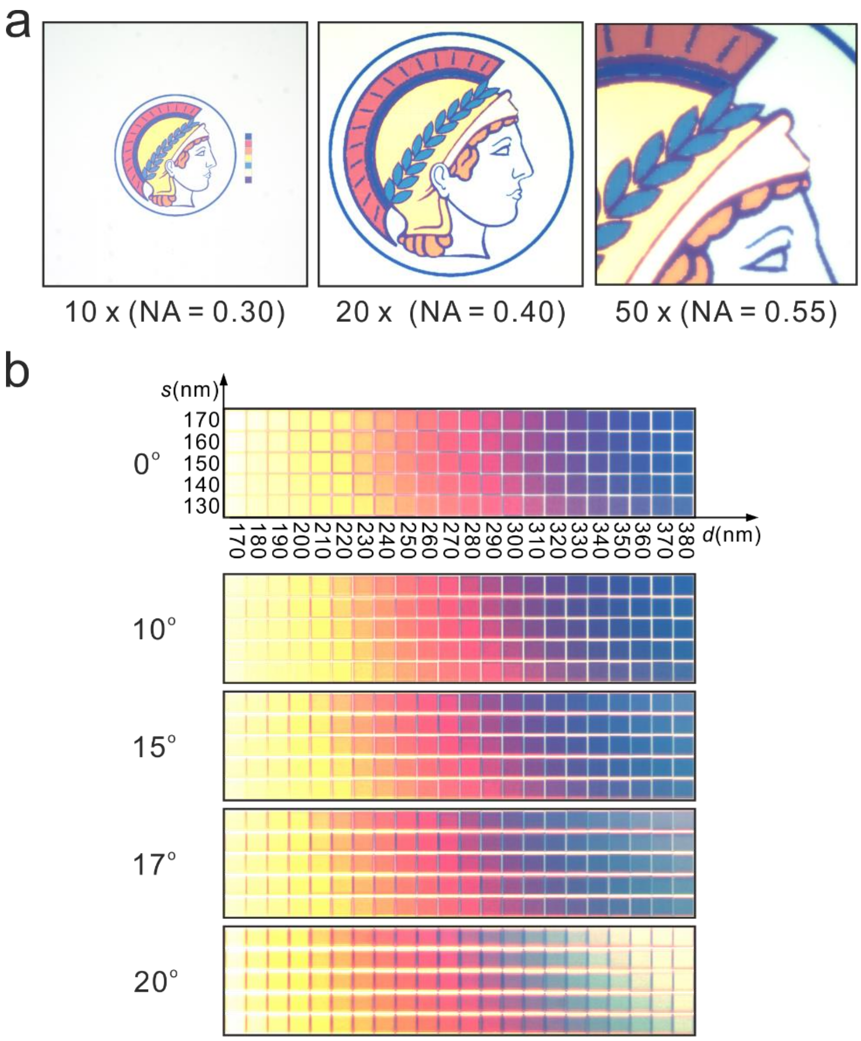

Supplementary Figure 10 | (a) Optical images of the Minerva logo under objectives with different NA values. (b) Optical images of one representative part of the colour palette illuminated under unpolarised light with the sample tilted at 0°, 10°, 15°, 17°, and 20°. Each of the rows in the tilted palette was imaged individually and arranged together to achieve a sharp, focused image. No significant colour changes are observed for tilting angles smaller than 17°. The colour changes are gradually observed from the colour squares with large pitches, when the tilting angle increases to 20°.

Predicted display images and colour scales during decryption/encryption

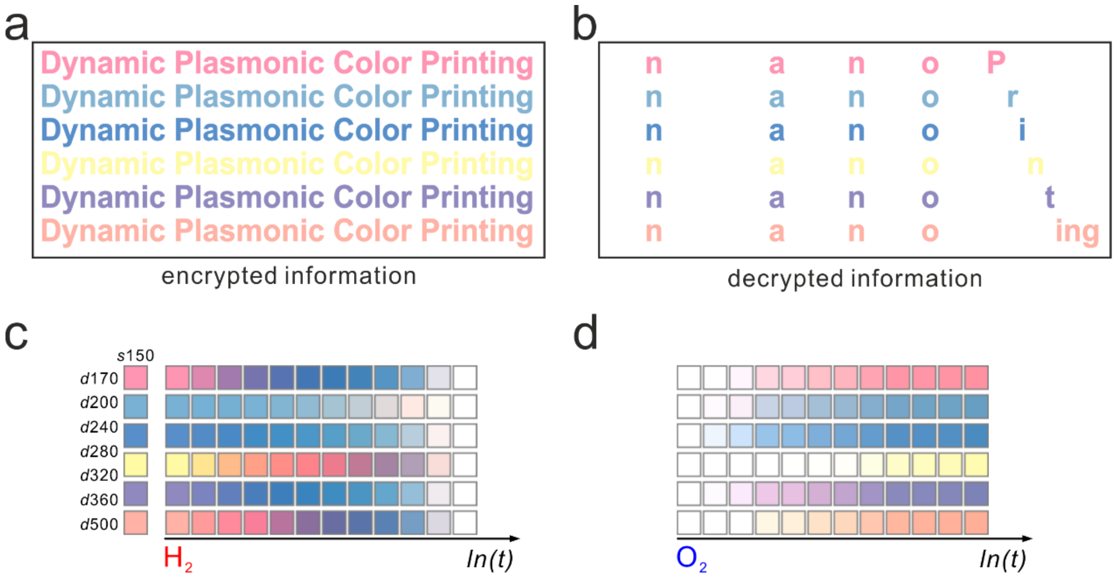

Supplementary Figure 11 | Predicted display images of Fig. 4c before (a) and after (b) hydrogenation. Colour scales during information decryption (c) and encryption (d).
